# HIV-1 and SUMOylation

**DOI:** 10.1186/1742-4690-10-S1-P106

**Published:** 2013-09-19

**Authors:** Guillaume Beauclair, Ali Saïb, Alessia Zamborlini

**Affiliations:** 1 CNRS/UP7 UMR7212, INSERM U944, Institut Universitaire d’Hématologie-Université Paris7 Diderot, Paris, 75010, France; 2 Conservatoire des Arts et Métiers, Paris, 75003, France

## Background

The HIV virus hijacks cellular machineries to replicate. Post-translational modifications, like acetylation, phosphorylation or ubiquitination, are no exception. We are interested in studying the interplay between HIV and the SUMOylation pathway.

SUMOylation occurs via an enzymatic cascade requiring an E1 activating enzyme (SAE1/SAE2 heterodimer) and an E2 conjugating enzyme (Ubc9). This modification is reversible thanks to SUMO isopeptidases (SENPs). Despite Ubc9 alone being sufficient to transfer SUMO moieties to acceptor lysine residues *in vitro*, E3 SUMO ligases promote specificity and/or efficiency of SUMOylation *in vivo.* So far, several E3 SUMO ligases have been characterized, such as nuclear pore complex component Ran-binding protein 2 (RanBP2) and protein-inhibitor of activated STAT (PIAS) family proteins.

We have recently shown that HIV integrase, the viral enzyme that is responsible for viral genome integration into host cellular chromosome, is SUMOylated, and that virions harboring a SUMOylation-defective integrase are less infectious than WT viruses. Our data suggest that this modification is important for efficient viral replication.

## Materials and methods

To detect protein SUMOylation, cells expressing His-tagged SUMO protein together with the protein of interest (HIV integrase) were lysed in denaturing conditions followed by enrichment on NiNTA colum. Bound proteins were eluted by boiling in Laemmli sample buffer supplemented with imidazole. Intracellular localization was studied by Duo LINK approach. Protein-protein interactions were analyzed by immunoprecipitation and GST pull-down assays.

## Results and conclusions

To better characterize the involvement of SUMOylation during HIV replication, we are currently studying the role of E3 SUMO ligases and SENPs in the conjugation of SUMO to integrase. We established that HIV integrase interacts with PIAS family member proteins and we showed that integrase and PIAS colocalise. Moreover, we demonstrate that PIAS1 and PIAS4, but not other PIAS family memebers, increase whereas SENP1 decreases integrase SUMOylation. We also analyzed the effect of PIAS over-expression in virus-producing or target cells and do not find a significant variation of viral infectivity. Interestingly, during HIV infection we show a decrease of the expression levels of one of the members of PIAS family. Experiments are ongoing to elucidate the mechanisms underlying this interplay between HIV and the SUMOylation pathway.

